# Comparison of the Effectiveness of New Material Diapers versus Standard Diapers for the Prevention of Diaper Rash in Chinese Babies: A Double-Blinded, Randomized, Controlled, Cross-Over Study

**DOI:** 10.1155/2018/5874184

**Published:** 2018-06-24

**Authors:** Chao Yuan, Rinko Takagi, Xue-Qiu Yao, Ya-fei Xu, Koichi Ishida, Haruko Toyoshima

**Affiliations:** ^1^Shanghai Skin Disease Hospital, No. 1278 Baode Road, Shanghai 200443, China; ^2^Sanitary Products Research, Kao Corporation, 2606 Akabane, Ichikai-machi, Haga, Tochigi 321-3497, Japan; ^3^Kao (China) Research and Development Center Co., Ltd., No. 623 Ziri Road, Minhang District, Shanghai 200241, China

## Abstract

**Background:**

Diaper rash, also known as diaper dermatitis (DD), is a very common skin condition in infants, and use of disposable diapers with breathable materials is an effective approach for the management of diaper rash. In China, new material diapers and standard diapers are currently the two most commonly used disposable diapers. This study aimed to compare the effectiveness of new material diapers versus standard diaper for the prevention of diaper rash in Chinese babies.

**Methods:**

A total of 80 eligible babies admitted to Shanghai Skin Diseases Hospital during the period from June through July, 2016, were enrolled and randomized into two groups. Babies in Group A (*n* = 41) used the new material diapers, and babies in Group B (*n* = 39) used standard diapers. Two weeks after the use of the diaper, the babies used the alternate product for the next 2 weeks. Skin conditions were assessed on the front and back waist, right and left buttock, pubic region, anal region, and right and left groin using a 6-point scoring system based on four parameters in 0, 2, and 4 weeks after use of the diapers.

**Results:**

There were changes of the mean skin assessment score in each of the six regions after the use of the diapers. There were significant differences, in the mean skin assessment score of the front waist in Group A between weeks 2 and 4 (*P* = 0.006) and in Group B between weeks 0 and 2 (*P* = 0.004), and no significant differences were detected in the mean skin assessment score of the back waist and buttock in both Group A and Group B on weeks 0, 2, and 4. A higher mean skin assessment score of the pubic region was assessed in Group A on week 4 than on week 2 (*P* = 0.036), with a higher score seen on week 2 than on week 0 (*P* = 0.048), while no significant differences were found in Group B among weeks 0, 2, or 4. There was a higher mean score of the anal region assessed in Group A on week 2 than on week 0 (*P* = 0.01), while a higher mean score was found in Group B on week 2 than on weeks 0 (*P* = 0.036) and 4 (*P* = 0.01). In addition, a higher mean skin assessment score of the groin was detected on week 2 than on week 0 in both Group A (*P* = 0.00001) and Group B (*P* = 0.0001).

**Conclusion:**

The new material diaper is superior to the standard diaper for the prevention of diaper rash in Chinese babies.

## 1. Introduction

Diaper rash, also known as diaper dermatitis (DD), is inflammation of the skin that appears on the skin under a diaper, notably in neonates and infants [1]. Diaper rash predominantly occurs in infants and children aged less than 2 years, with the highest incidence found in infants at the age of 7 to 12 months [2]; however, this concern may be also seen in adults who wear diapers for incontinence of urine or stools [3]. As one of the most common skin conditions in infants, diaper rash was reported to account for approximately 20% of all pediatric office visits [4, 5]. Although the disorder is not usually life threatening, it may cause erythema and itching in the affected areas for infants and children and may cause significant distress for parents [6, 7].

Currently, the treatments for diaper rash mainly include minimizing diaper use and using disposable diapers with breathable materials, barrier creams, and using the mild topical cortisones or antifungal agents if necessary [8–10]. In China, new material diapers, which have high breathable layer and high absorbent layer and airy wavy soft surface, and standard diapers are the two most popular brands of disposable diapers at present. New material diaper is constituted from three air-through structures, including the high breathable layer, the block-shaped superabsorbent sheet, and the soft airy inner wavy surface. The inner wavy surface makes the space between the diaper and skin because of the wavy shape, and the contact between the surface and skin is reduced by half of that of the flat surface like standard diaper. The block-shaped absorbent sheet has also many air-through spaces inside. These air-through structures facilitate expelling moisture and stuffiness from the insides of the diaper and achieved high breathability and help providing long-lasting dryness for babies. The inner wavy surface traps and holds stool, preventing it from dispersing, while the superabsorbent sheet instantly draws in and locks away urine and soft stool, leaving the diaper surface drier. It is estimated that this new material diaper makes it gentle on baby's delicate skin. The aim of this study was to evaluate the efficacy of the “new material diaper” with newly improved technological features for mild diaper rash as compared with the standard diaper.

## 2. Subjects and Methods

### 2.1. Ethical Approval

This study was approved by the Ethical Review Committee of Shanghai Skin Diseases Hospital (permission no. 2016-001). Written informed consent was obtained from the participants' parents following a detailed description of the purpose and potential benefits of the study.

### 2.2. Study Subjects and Grouping

Babies at ages of 3 to 24 months were recruited to Shanghai Skin Diseases Hospital (Shanghai, China) during the period between June and July, 2016, through surveyor's description, registration in network platforms, and screening in infant test banks. The inclusion criteria included (1) 3-to-24-month-old babies that used more than 3 disposable diapers per day; (2) the parents willingness to sign the informed consent to participate in the study; and (3) babies without diaper rash or very mild rashes (Score 0–2, score is defined according to the following section or Table 1 (Assessment of Skin Conditions) [11]), and (4) all babies that had used the same types of standard and new diapers in the past with those used in the present study. Those with the following criteria were excluded from the study: (1) babies with atopic dermatitis or other skin disease; (2) babies with systemic illness; (3) babies undergoing treatment for skin disorders; (4) babies that experienced diarrhea during screening, and (5) babies that used any diaper rash cream during the study period. Finally, a total of 80 babies that met the inclusion and exclusion criteria were enrolled in the study.

To assess the efficacy of the diaper for prevention of diaper rash, a double-blinded, randomized, controlled, cross-over study was therefore designed [12]. The eligible babies without diaper rash were randomized into two groups according to the skin evaluation to allow no significant difference in the skin conditions score when they were assessed at baseline. Babies in Group A used the new material diapers, and babies in Group B used standard diapers. Two weeks after the use of the diaper, the babies used the alternate product for the next 2 weeks. Each infant stayed in the hospital for 2 to 3 hours in the company of their parents and returned to the hospital for follow-up visits 2 and 4 weeks after discharge.

According to the inclusion criteria, babies without diaper rash or very mild rashes (Scores 0 to 2) were enrolled. Therefore, there were no babies withdrawing from the study due to adverse events.

### 2.3. Assessment of Skin Conditions

Baby's diaper areas were graded by the same two dermatologists in the same lighting condition for each visit. After the babies were transferred to a laboratory, the diapers were firstly uncovered, and the babies were placed at 18 to 22°C and in a relative humidity of 40% to 60% for at least 30 min prior to skin assessment. If the baby cried or was receiving breast-feeding, skin assessment was completed after the baby was quiet or following the breast-feeding. Skin conditions were assessed on the front and back waist, right and left buttock, pubic region, anal region, and right and left groin using a 6-point scoring system based on four parameters in 0, 2, and 4 weeks after use of the diapers (Table 1) [11].

### 2.4. Statistical Analysis

All measurement data were described as mean ± standard deviation (SD), and all categorical data were expressed as proportions. The intragroup difference of skin condition scores was compared with Wilcoxon test, and the intergroup difference of skin condition scores were tested for statistical significance using Mann–Whitney *U* test. All statistical analyses were performed using Excel statistics 2012 (Social Survey Research Information; Tokyo, Japan) and the statistical software SPSS version 17.0 (SPSS, Inc.; Chicago, IL, USA), and a *P* value of < 0.05 was considered statistically significant.

## 3. Results

### 3.1. Subject Characteristics

A total of 211 babies were recruited, and 131 babies were excluded. Finally, 80 eligible babies were randomized into the two groups. There were 41 babies in Group A, including 18 females and 23 males, and 39 babies in Group B that completed the tests, including 18 females and 21 males. The babies in Group A had a mean age of 15 months (range, 4 to 24 months), and the babies in Group B had a mean age of 15 months (range, 5 to 24 months). There were no significant differences between the two groups in terms of age and gender distribution and mean daily stool frequency (all *P* values > 0.05) (Table 2).

### 3.2. Skin Assessment Score

In this study, the skin conditions were scored in the front waist, back waist, buttock, pubic region, anal region, and groin before and after the use of diapers. Overall, more babies with improved skin assessment scores were observed in using the triple-layer air-through diaper than in standard diaper, notably in the buttock and anal region.

There were changes of the mean skin assessment score in each of the six regions. In the anal region, there was a remarkable reduction in the skin assessment skin in Group B after the use of the new material diapers, notably 2 and 4 weeks, and, in the front waist, the skin assessment score decreased significantly in both Groups A and B after the use of the new material diapers.

**Figure 1:**
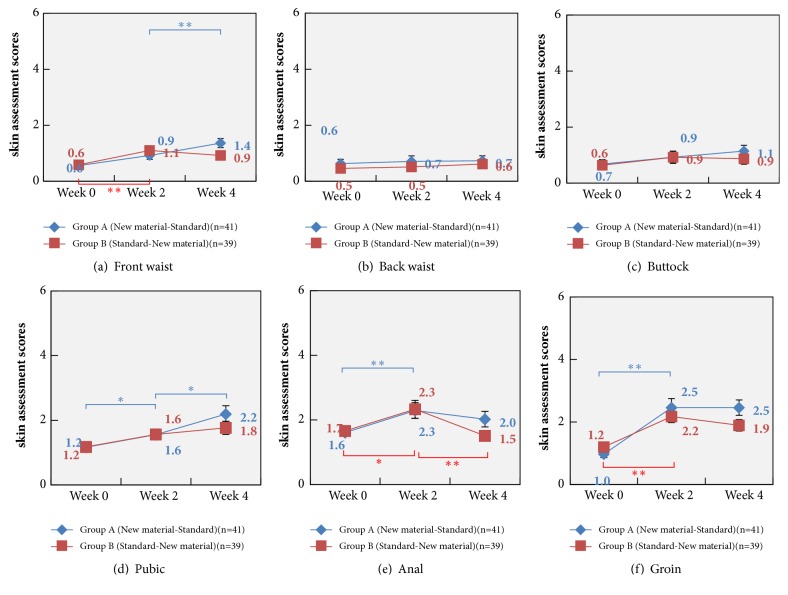
Skin assessment scores of the front (a) and back waist (b), the buttock (c), pubic region (d), anal region (e), and the groin (f) using a 6-point scoring system based on four parameters 0, 2, and 4 weeks after use of the diapers (^*∗*^*P* < 0.05; ^*∗∗*^*P* < 0.01).

There were no significant differences in the skin assessment score between Groups A and B (Table 3). The mean skin assessment score of the front waist showed an increasing tendency in Group A on weeks 2 and 4; and it increased on week 2 and then decreased on week 4 in Group B. Wilcoxon test revealed significant differences in Group A between weeks 2 and 4 (*P* = 0.006) and in Group B between weeks 0 and 2 (*P* = 0.004) (Figure 1(a)). There were no significant differences detected in the mean skin assessment score of the back waist and buttock in both Group A and Group B on weeks 0, 2, and 4 (Figures 1(b) and 1(c)). The mean skin assessment score of the pubic region showed an increasing tendency in both Groups A and B on weeks 0, 2, and 4 and a higher mean skin assessment score was assessed in Group A on week 4 than on week 2 (*P* = 0.036), with a higher score seen on week 2 than on week 0 (*P* = 0.048), while no significant differences were found in Group B among weeks 0, 2, or 4 (Figure 1(d)). The mean skin assessment score of the anal region increased on week 2 in both Group A (*P* = 0.01) and Group B (*P* = 0.036) and then decreased on week 4 in Group B with significant differences (*P* = 0.01) (Figure 1(e)). In addition, a higher mean skin assessment score of the groin was detected on week 2 than on week 0 in both Group A (*P* = 0.00001) and Group B (*P* = 0.0001) (Figure 1(f)).

Our data show that there are no significant differences in the mean skin assessment score at some sites before and after the use of new material and standard diapers; however, the results of this 4-week cross-over study demonstrate that the new material diaper is superior to the standard diaper for preventing diaper rash in front waist, pubic region, and anal region.

## 4. Discussion

With the increasing developments in noninvasive skin measuring, many researches have showed us that the infant skin is in a developmental stage structurally up to 12 months of age, paralleling skin functional and developmental maturation [13–15]. The nondiapered skin has low high transepidermal water loss (TEWL) at birth in full-term infants and increases over time during the first year [16]. In the development time of baby skin barrier, we should pay more attention to the skin care of them, and diaper using is one of the key points among them.

In old time and small cities, cloth diapers had been most widely used in Chinese infants [17, 18]; however, the use of cloth diapers was found to greatly cause the likelihood of developing diaper dermatitis in children, notably in the perianal and intertriginous regions [19]. Then, disposable diaper was introduced and now is widely used in China [20–22], which was found to promote consolidated nighttime sleep and positive mother-infant interactions [23]. Most importantly, the use of disposable diapers has been proved to be effective in preventing the emergence of diaper rash [24–26], because (1) excrement, such as urine and poo, does not adhere to skin easily, especially for the high quality materials and (2) the inside of a disposable diaper space cannot be easily filled with humidity if the mother changes it in time.

A standard disposable diaper is composed of a top sheet, an absorbent core, gathers, and a breathable sheet. Following the use of the standard disposable diaper, skin may be damped by sweat or pee and facilities spread and adherence to skin, which irritates skin, thereby resulting in the development of diaper rash. The baby skin barrier is not healthy enough in a developmental stage. Humidity, nonbreathability, adherence of excrements to skin, friction, and relatively weak infant skin barrier are considered to be the leading contributors to the high incidence of DD. The new material diaper is a triple-layer air-through diaper, which is composed of (1) a wavy top sheet, which reduces the area of contact, allows free air-through, and has less diffusion of poo and a soft surface; (2) a block-shaped absorbent core, which may absorb urine in a dot-shaped manner, allows free air-through and does not expand in the presence of poo; (3) a breathable back sheet, which has a high breathability; and (4) a soft-fit structure, which provides adequate space and comfort for babies' activities.

It has been shown that the materials and design of the diaper exhibit an important impact on babies' skin barrier functions [27]. Comparison of the diffusion of water poo, skin hydration, and breathability showed that the new material diaper, unlike the standard diaper, cannot make excrement adhere to skin easily, and it cannot be easily filled with humidity. Currently, the new material and the standard material are the two most commonly used types of disposable diapers in China. However, there have been no studies comparing the effectiveness of these two disposable diapers for the prevention of diaper rash until now. This double-blinded, randomized, controlled, cross-over study was therefore designed to compare the effectiveness of new material versus standard diapers for the prevention of diaper rash in Chinese babies.

Interestingly, we found similar behavior and no significant difference in the mean skin assessment score of the anal region between Groups A and B, and the mean score increased on week 2 relative to week 0 in both groups. However, the mean score decreased from 2.3 to 2 in Group A and from 2.3 to 1.5 in Group B, indicating the use of the new material diapers improves the skin conditions on the anal region. At the end of June in Shanghai, 2016, the mean air temperature was relatively low, with a mean daily air temperature of below 25°C, and the relative humidity was approximately 40%. However, the air temperature was over 30°C on July, which obviously affected the skin barrier function [28]. Therefore, we found higher skin assessment scores in babies on week 2. Then, the reduction in air temperature and humidity caused improvements of the skin barrier function on weeks 2 to 4. Even if in atrocious weather, the new material diapers still exhibited satisfactory protection for the perianal region, which easily becomes humid and is the most common region that urine or poo adheres to. However, further studies to determine the specific diaper-skin factors responsible for the findings are warranted.

The present study has some limitations. (1) The study size is small. Further studies recruiting more subjects are required to validate the findings from the current study and more skin biophysical parameters could be noticed to learn more about the baby skin barrier changes in diaper areas. (2) Since climate conditions affect skin barrier functions, further studies should be conducted in seasons with appropriate air temperature and humidity, which may avoid the effect of confounding factors like external environments and climate factors, on skin irritation.

In summary, the results of the present study demonstrate that the new material diaper is superior to the standard diaper for the prevention of diaper rash in Chinese babies.

## Figures and Tables

**Table 1:** Grading criteria for diaper rash.

Score	Erythema/Edema	Papula/pustule	Immerse/erosion	Desquamation
0	Absent	Absent	Absent	Absent
1	Very slight erythema, area < 2%	Only one site	Slight immerse, area < 2%	Slight desquamation, area < 2%
2	Very slight erythema, area (2-10%) or slight erythema, area < 2%	Discrete papula, 2–5 sites	Slight immerse, area (2–10%)	Slight desquamation, area (2–10%)
3	Very slight erythema, area > 10% or slight erythema, area (2–10%) or obvious erythema < 2%	Discrete papula, area < 10%	Slight to moderate immerse, area > 10% or slight erosion, area < 2%	Slight to moderate desquamation, area > 10%
4	Slight erythema, area (10 – 50%) or obvious erythema, area < 2% with edema	Moderate papula, area (10–50%) or pustule (0 to 5 sites)	Moderate immerse, area (10–50%) or slight erosion, area (2–10%)	Moderate desquamation, area (10–50%)
5	Obvious erythema, area > 50% or obvious erythema, area (2–10%) with edema	Moderate to severe papula, area > 50% or pustule (over 5 sites)	Moderate to severe immerse, area > 50% or moderate erosion, area > 10%	Moderate to severe desquamation, area > 50%
6	Obvious erythema, area > 10 % with edema	Large area confluent papula or large pustule/ blister	Severe erosion, area > 50% or ulcer, necrosis	Severe desquamation

**Table 2:** Age, gender distribution, and daily stool frequency of the study subjects.

Characteristics		Group A	Group B
Age	Mean age (mean ± SD, months)	15.3 ± 6.3	15 ± 6.1
	No. of ≤6 months	2	3
	No. of 6 to 12 months	13	11
	No. of ≥12 months	26	25
Gender	No. of men	23	21
	No. of women	18	18
Mean daily stool frequency	Weeks 0 to 2	1.3	1.1
	Weeks 2 to 4	1.3	1.0

Total		41	39

**Table 3:** Skin assessment score at various sites (mean ± SE).

Region	Group	Week 0 (baseline)	Week 2	Week 4
Front waist	A	0.6 ± 0.1	0.9 ± 0.1 (new diaper)	1.4 ± 0.2^*∗∗*^ (standard diaper)
	B	0.6 ± 0.1	1.1 ± 0.1^*∗∗*^(standard diaper)	0.9 ± 0.2 (new diaper)
Back waist	A	0.6 ± 0.2	0.7 ± 0.2 (new diaper)	0.7 ± 0.2 (standard diaper)
	B	0.5 ± 0.2	0.5 ± 0.1 (standard diaper)	0.6 ± 0.2 (new diaper)
Buttock	A	0.7 ± 0.2	0.9 ± 0.2 (new diaper)	1.1 ± 0.2 (standard diaper)
	B	0.6 ± 0.2	0.9 ± 0.2 (standard diaper)	0.9 ± 0.2 (new diaper)
Pubic region	A	1.2 ± 0.1	1.6 ± 0.2^*∗*^ (new diaper)	2.2 ± 0.3^*∗*^ (standard diaper)
	B	1.2 ± 0.1	1.6 ± 0.1 (standard diaper)	1.8 ± 0.2 (new diaper)
Anal region	A	1.6 ± 0.1	2.3 ± 0.2^*∗∗*^ (new diaper)	2.0 ± 0.2 (standard diaper)
	B	1.7 ± 0.1	2.3 ± 0.3^*∗*^ (standard diaper)	1.5 ± 0.1^*∗∗*^ (new diaper)
Groin	A	1.0 ± 0.1	2.5 ± 0.3^*∗∗*^ (new diaper)	2.5 ± 0.2 (standard diaper)
	B	1.2 ± 0.1	2.2 ± 0.2^*∗∗*^ (standard diaper)	1.9 ± 0.2 (new diaper)

^*∗*^ *P* < 0.05 versus week 0; ^*∗∗*^*P* < 0.01 versus week 0.

## Data Availability

All data included in this study is available upon request by contact with the corresponding author.
